# Warming altered the effect of cold stratification on the germination of *Spartina alterniflora* across climatic zones in its invasive range

**DOI:** 10.3389/fpls.2024.1491275

**Published:** 2024-11-26

**Authors:** Fujia Wu, Xincong Chen, Yangping Guo, Wenwen Liu, Yihui Zhang

**Affiliations:** Key Laboratory of the Ministry of Education for Coastal and Wetland Ecosystems, College of the Environment and Ecology, Xiamen University, Xiamen, Fujian, China

**Keywords:** cold stratification, invasion, latitude, seed germination, *Spartina alterniflora*, warming, common garden

## Abstract

**Introduction:**

Cold stratification has a pronounced influence on seed germination, climate change is altering cold stratification regimes across climatic zones. Therefore, it is urgent to explore how seed germination from different geographic provenances responds to these changes. The invasive plant Spartina alterniflora spans three climatic zones along the Chinese coast, such distribution provides a natural temperature gradient to explore how warming alters the effects of cold stratification on germination.

**Methods:**

Spartina alterniflora seeds were collected from nine locations across three climatic zones in China from September to November in 2021. Seeds were planted in three common gardens with three latitude gradients of 21 °N, 28 °N, and 38 °N, after 0-month and 4-month cold stratification at 4 °C in November 2021 and March 2022, respectively. Each common garden simulated the natural temperature conditions and shield the plants from rain.

**Results:**

Results showed that cold stratification led to explosive germination and rapidly reaching a plateau, shortened the germination time and improved the final germination rate. These effects were magnified from the high-latitude garden to the low-latitude one (i.e., warming). And the interactive effect of cold stratification and warming varied among provenances. For the subtropical and temperate provenances, the improvement in germination rate induced by cold stratification gradually increased from high-latitude garden to low-latitude one, while for tropical provenances, this difference progressively decreased. Discussion: Thus, our results indicated that subtropical and temperate provenances may migrate northward for adequate low temperatures to ensure high germination rate, because cold stratification can alleviate the negative impacts of warming on germination. For the tropical provenances, warming also suppressed the advantage that cold stratification provides in enhancing the germination rate, which may hinder their further spread southward. Our study contributes to understanding the responses of vegetation germination and recruitment across different climatic zones under global warming, providing insights for the distribution of cosmopolitan species and the management of exotic species.

## Introduction

Climate warming affects the life history of plants, of which the germination period is considered to be the most sensitive to stochastic environments, with lasting effects on individual adaptation, population persistence, and species distribution (Post et al., 2008; Springthorpe and Penfield, 2015; Collins et al., 2021; Keller and Shea, 2021). Seed germination marks the beginning of a plant’s life history and its characteristics, such as germination time and rate, reflect the seed’s adaptability to environments (Donohue et al., 2010; Huang et al., 2016). Plants adjust their germination characteristics to optimize survival and reproduction (Donohue et al., 2005; Gremer et al., 2020b). Cold stratification is a crucial physio-ecological processes for seed germination in most temperate regions (Baskin and Baskin, 1988), enabling seed to germinate under appropriate conditions (Steadman and Pritchard, 2004; Cavieres and Sierra-Almeida, 2018). Cold stratification is defined as the way to store mature seeds on water-moistened substrates in darkness at low temperature (typically around 4°C) (García-Fernández et al., 2015; Hayasaka et al., 2020). With the increase of temperature, there is an adaptive shift in the response of seeds to cold stratification (Bernareggi et al., 2016). For instance, some plants may gradually adapt to warmer climates by adjusting the germination time (Walck et al., 2011; Boheemen et al., 2019), while others may migrate through niches in search of more suitable habitats (Lustenhouwer et al., 2018). Temperature is the primary factor regulating seed germination, and climate change may alter or disrupt germination characteristics in different regions under new climate (Walck et al., 2011; Zhou and He, 2020). However, it is still unknown how the effects of cold stratification on seed germination characteristics in broad geographic areas would change under climate warming.

The heterogeneity of environmental conditions in different geographical regions along latitude leads to different germination characteristics and different responses to cold stratification (Chamorro et al., 2018; Cheng et al., 2022). In temperate regions, longer and cooler winters result in deeper seed dormancy (Gremer et al., 2020a; Kuroda and Sawada, 2022), and seeds often delay germination to avoid frost damage (Center et al., 2016). In this case, cold stratification becomes a necessary physiological process to drive seed germination, and the germination time directly reflects the adaptability to current environmental conditions (Donohue et al., 2005). Whereas, plants in tropical regions with warmer and shorter winter may not meet an obvious stratification process (Spindelböck et al., 2013). Climate warming could change the regimes of cold stratification along environmental gradients (Orru et al., 2012; Kuroda and Sawada, 2022). However, seeds from different geographic regions are facing with different extent of warming, many studies indicate that high-latitude regions are more sensitive to climate changes than other regions (Kirwan et al., 2009; Cohen et al., 2014; Leblans et al., 2017). Understanding the potential alternation in germination in various geographic regions is crucial for predicting adaptability and distribution of cosmopolitan species under warming.

Exotic invasive plants, which often span wide latitudinal gradients, and benefit from climate warming (van Kleunen et al., 2010; Wu et al., 2017; Keller and Shea, 2021). However, responses of invasive plants’ germination to cold stratification across latitudes are limited. *Spartina alterniflora* is a widely distributed global invasive plant, native to the Atlantic and Gulf Coasts of the United States (Castillo et al., 2014; Zhang et al., 2023). Its rapid spread in the new habitats is related to the adaptability of the seed during the germination stage (Biber and Caldwell, 2008; Liu and Zhang, 2021). In its native range, germination traits show local adaptation along latitudinal gradient with genetic variation basis, primarily related to temperature (Mooring et al., 1971; Seneca, 1974). In its invaded area, growth chambers studies also suggested that temperature variations along latitude drove changes in seed germination characteristics and influenced the geographical distribution of *S. alterniflora* (Liu and Zhang, 2021; Cheng et al., 2022). However, natural habitats are complex, which poses a challenge to fully simulate these environments in growth chambers. Under climate warming, temperature changes in different geographical regions may have significant effects on germination characteristics via changing the cold stratification regimes. Researches on the response of *S. alterniflora* germination to varying temperature conditions is crucial for better understanding its invasive dynamic under climate warming. Observation and experimental warming methods are main approaches to empirically examine the effects of warming on seed germination. Nevertheless, the results of observational studies are often difficult to generalize across different times and spaces, while experimental studies, typically conducted on smaller spatial or temporal scales, frequently use stepwise temperature increases that may not fully represent natural conditions. Common garden experiments, which compare seed germination across sites with contrasting temperatures, such as latitudinal gradient, provide crucial insights into how temperature influences germination traits (Moloney et al., 2009; Frenne et al., 2013).

In this study, seeds from nine locations of *S. alterniflora* were collected across a latitudinal range in China. These seeds were planted in three common gardens spanning three latitudinal gradients (low-, mid- and high-latitude) after cold stratification (4°C) for 0-month and 4-month. The high-, mid-, and low-latitude common gardens are respectively located in Shandong, Zhejiang, and Guangdong province, and the high-/low-latitude common gardens are close to the north/south boundaries of the distribution of *S. alterniflora* in China. These three common gardens span three climatic zones and nearly 20 latitudes, with different temperature conditions (**Supplementary Table S3**). Macroclimatic variation along latitudinal gradient provides an excellent natural laboratory to investigate the role of temperature and the effects of climate warming on seed germination (Frenne et al., 2013). We aimed to answer: (1) What is the specific effect of cold stratification on germination rate and timing of *S. alterniflora* across different climates? (2) How does climate warming alter these effects in various climatic zones? (3) Do the effects of cold stratification and warming on germination vary among provenances from different climatic zones? The answers would be beneficial for understanding the recruitment and predicting the spread of *S. alterniflora* provenances inhabiting different climatic zones in the scenario of climate warming.

## Materials and methods

### Study species

*Spartina alterniflora* Losiel., native to North America and the Gulf Coast of the Mexico, is a global invasive plant in coastal wetlands (Daehler and Strong, 1994; Zhang et al., 2023). In 1979, three ecotypes of *S. alterniflora* were introduced to Fujian, China, from three different geographical regions in the United States: North Carolina (34.72°N), Georgia (31.47°N), and Florida (27.70°N) (Xu and Zhuo, 1985). *Spartina alterniflora* can reproduce both by seeds and vegetative fragmentation, and sexual reproduction is conducive to long-distance transmission (Daehler and Strong, 1994). Over the past 40 years since its introduction, *S. alterniflora* has already rapidly spread across a 20-degree latitudinal range through both artificial planting and natural dispersal, spanning tropical, subtropical and temperate regions (An et al., 2007; Zhang et al., 2023; Zhao et al., 2023).

### Seed collection and pre-treatment

**Figure 1:**
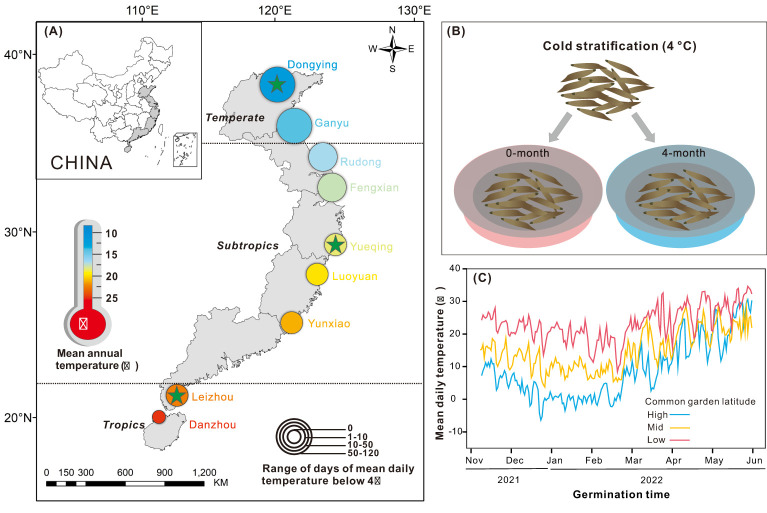
**(A)** *Spartina alterniflora* seed collection locations in China (circle), three common garden sites (star), mean annual temperature (color) and range of days of mean daily temperature below 4 °C (circle size) variation across latitude. **(B)** Cold stratification for 0-month and 4-month. **(C)** Mean daily temperatures during the germination period in three common gardens.

Seeds of *S. alterniflora* were collected from nine locations with latitude intervals of 1~2° within the invasive range of China (ranging from 20°N to 38°N). (**Figure 1A**; **Supplementary Table S2**). We collected seeds from September to November in 2021, which represented the end of the growing season at all locations. *Spartina alterniflora* matures earlier at lower latitudes (Liu et al., 2016; Chen et al., 2021), therefore, seeds collection was conducted from south to north.

At each location, we worked at two sites, located 2-3 km apart. Five quadrats (0.5 × 0.5 m) were established per site, which were at least 30 m to ensure that they were from different clones, each quadrat considered a seed family. From each quadrat, at least 15 inflorescences were randomly collected, ensuring no herbivory or shattering. We distinguished between filled (embryo-containing) and unfilled (embryo-lacking) seeds by touch (Schmidt-Adam et al., 2002; Hayasaka et al., 2020).

In the past 10 years, seeds of *S. alterniflora* have experienced between 0 (Danzhou) and 114 (Dongying) days with mean annual temperature below 4°C, which indicates cold stratification time (**Figure 1A**; **Supplementary Table S2**). Before the germination experiments, the seeds were covered with 10 PSU seawater under darkness conditions. They were placed in a refrigerator at 4°C for 0-month and 4-month to explore the effect of cold stratification treatment on seed germination (**Figure 1B**).

### Multiple common garden germination experiments

To investigate the adaptation of germination characteristics of *S. alterniflora* from different latitudinal provenances to natural environments in different climate zones, we established common garden at three latitudes: low (21°N, Guangdong province), mid (28°N, Zhejiang province), and high (38°N, Shandong province), respectively located in tropical, subtropical, and temperate regions (**Figure 1A**). Because temperature is the primary factor regulating seed germination, and also the most critical factor to *S. alterniflora* development (Chen et al., 2021). To focus on the interactive effects between cold stratification and temperature, we minimized differences in other environmental conditions, such as precipitation. Each common garden simulated the natural temperature conditions and shield the plants from rain by covering the top of the common gardens with transparent plastic film. Additionally, the rain protection can keep seawater salinity stable in each pool. Nine provenances were also categorized into tropical regions (Leizhou, Danzhou), subtropical regions (Rudong, Fengxian, Yueqing, Luoyuan, Yunxiao) and temperate regions (Dongying, Ganyu) (**Figure 1A**; **Supplementary Table S2**). Climatic zones were divided according to the resource and environment science data center, aligning with the division in Qiao et al. (2019).

In each common garden, began with November 10^th^ 2021, we sowed the seeds that underwent 0-month stratification (**Supplementary Table S1**). Twenty seeds of each seed family from nine provenances were sown in a plastic bucket. Each plastic bucket has been uniformly filled with a mixture of 50% Jiffy peat substrate (Jiffy Products International BV, Moerdijk, Netherlands) and 50% vermiculite before the sowing. Seeds of each seed family were randomly sown on a grid plate (2 × 2 cm) with 4 × 5 hole in the center of each bucket, with one seed sown in each hole. After sowing, approximately 0.2 cm of soil was added to cover the seeds’ surfaces. Each seed family was considered as a replication of a provenance, and 10 seed families were separately arranged into 10 rectangular plastic pools (length: 110 cm, width: 90 cm, tall: 30 cm). The pools were filled with artificial salinity level of 10 PSU, to parallel with the soil surface level in the plastic buckets. Fresh water was added every other day to maintain salinity at 10 PSU. Each bucket had a 1-cm hole at the bottom for seawater exchange. In each common garden, began with March 09^th^ 2022, the seeds that underwent 4-month stratification were sown using the same method as the seeds that underwent 0-month stratification.

We monitored germination daily in the common gardens since November 10^th^ 2021. Because it is difficult to assess radicle emergence from the seed coat in soil, germination in this study was recorded when seeds emerged from the soil surface (Fenner and Thompson, 2005). By May 2022, most seeds had germinated, and no new germinations were observed for over a week by late May, thus germination experiment ended on May 31^th^ 2022. The cumulative germination rate, mean germination time, T_90%_ (the number of days to 90% of the final germination rate) and final germination rate were calculated for each quadrat to estimate germination capacity.

### Abiotic factors

To relate the germination characteristics of *S. alterniflora* to abiotic conditions, we collected the environmental data from provenance origins and germination process temperature. We calculated climate data on mean annual temperature (T_mean_), mean annual maximum temperature (T_max_), mean annual minimum temperature (T_min_) and annual number of growing degree days (mean daily temperature ≥10°C; AGDD) of each provenance from 2011 to 2021 from the UK weather data service center (http://rp5.ru). We also calculated the range of days of mean daily temperature below 4°C (D_below 4°C_) of each provenance from 2011 to 2021 (**Supplementary Table S2**). Additionally, a temperature logger (Onset, HOBO) was set 1 meter above ground at the center in each common garden to record temperature variations every 10 minutes. We calculated the mean daily temperature (T’_mean_), mean daily maximum temperature (T’_max_), mean daily minimum temperature (T’_min_), the number of growing degree days (mean daily temperature ≥10°C; GDD) and days of mean daily temperature below 4°C (D’_below 4°C_) from November 2021 to May 2022 in each common garden (**Figure 1C**; **Supplementary Table S3**).

### Data calculations and statistical analyses

The cumulative germination rate, mean germination time, final germination rate and T_90%_ (the number of days to 90% of the final germination rate) values are calculated as follows:


(1) $$\text{Cumulative germination rate}\;\left(\%\right)=\sum_{i=1}^{d}\frac{Ni}{N}\times 100$$

(2) $$\text{Mean germination time }\left(\text{d}\right)=\sum_{i=1}^{d}T_{i}N_{i}/\sum_{i=1}^{d}N_{i}$$

(3) $$\text{Final germination rate }\left(\%\right)=\frac{n}{N}\times 100$$

(4) $${\text{T}}_{90\%}\left(\text{d}\right)=D_{i=}90\%–D$$

In the formulas above, *d* signifies the total duration of the experiment in days, *N_i_* stands for the number of seeds that germinated on a specific day *i*, *N* is the total number of seeds tested in each seed family, *T_i_*_’_ indicates the number of days that germinated on a specific day *i* from the date of sowing, and *n* is the total number of seeds germinated at the end of the experiment, *D_i=_*_90%_ is date on which the germination rate reaches 90% of the final germination rate, *D* is the date of sowing (Carpenter et al., 1994; Ranal and de Santana, 2006; Soltani et al., 2015).

In the three common gardens, 5990 seeds were successfully germinated. We plotted the cumulative germination rate over time for each provenance under different cold stratification treatments within each common garden. To identify the peak germination timing under different cold stratification treatments within each common garden, we employed the “density ridge” to estimate the probability density distribution of germination timing. The data utilized the germination timing of germinated seeds (n = 581-1159), with areas of higher density indicating peaks in germination timing. We utilized the Wilcoxon test to assay the number of days to 90% of the final germination rate (T_90%_) between two cold stratification treatments within each common garden. In addition, we conducted linear regression analyses to assess the relationship between mean germination time and the latitude of seed provenance under different stratification treatments for each common garden. To determine whether significant differences exist in the final germination rate (average across all seed from different provenances) between two cold stratification treatments within each common garden, we employed the Wilcoxon test. To further explore the response of final germination rate to cold stratification of seed provenance from different climate regions within each common garden, we averaged the values of seed provenances within the same climate region. We also utilized linear regression analysis to examine the relationship between the final germination rate and the latitude of seed provenance under different stratification treatments for each common garden. To validate the impacts of common garden site, cold stratification time, and latitude of seed provenance, as well as their interactions on germination traits, we utilized linear mixed effects models with the “lme4” package (function “lmer”) (Bates et al., 2015). This model considered mean germination time and final germination rate as response variables, common garden site, cold stratification time, and latitude of seed provenance as fixed factors, while considering provenance as a random effect. To quantitatively analyze the contributions of environmental climate variables from provenance origin and the germination process in the common garden to the mean germination time and final germination rate, we employed the “rdacca.hp” package to hierarchical partitioning of seed’s origin environmental climate variables and environmental climate variables during the germination process in the common garden (Liu et al., 2023). This allowed us to calculate the individual effects of each group of explanatory variables on explanatory variation (adjusted *R* ^2^) in canonical analysis. All statistical analyses were conducted using R version 4.2.2.

## Results

### Variation in cumulative germination rate

**Figure 2:**
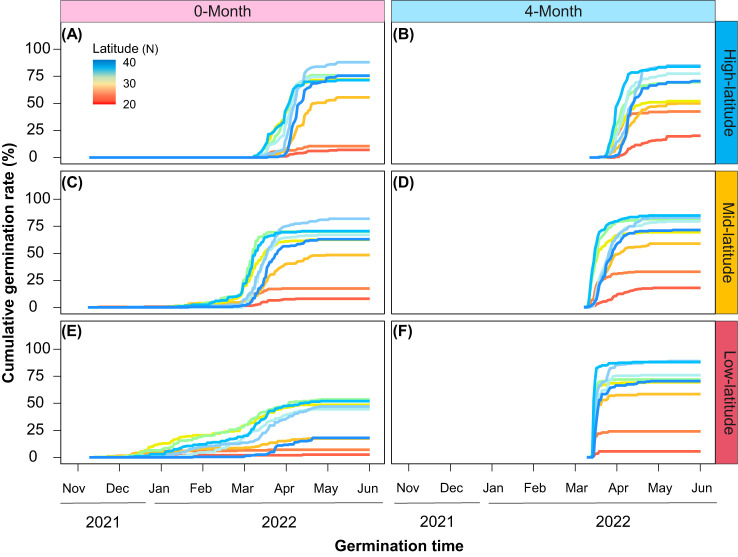
Cumulative germination percentage of different provenances under different stratification time (**A, C, E**: 0-month; **B, D, F**: 4-month) and in different common gardens (**A, B**: high-latitude; **C, D**: mid-latitude; **E, F**: low-latitude).

Cold stratification (4-month) significantly changed the cumulative germination rates, the germination was explosive and reached a plateau rapidly (**Figure 2**). Additionally, the effect of cold stratification on cumulative germination rates differed across gardens. With increased temperature, the curve trends of cumulative germination rates became much steeper in the low-latitude garden than the other ones (**Figures 2B, D, F**). From the high-latitude garden to the low-latitude one, it took 36, 25, and 12 days, respectively, to reach 90% of the final germination rate (**Supplementary Figure S1**). All provenances although showed similar responses under cold stratification in different gardens, the extent of variation in cumulative germination rates were different. Temperate provenances had higher and faster germination rates than the others. And from the high-latitude garden to the low-latitude one, such differences were gradually magnified (**Figures 2B, D, F**).

### Variation in germination time

**Figure 3:**
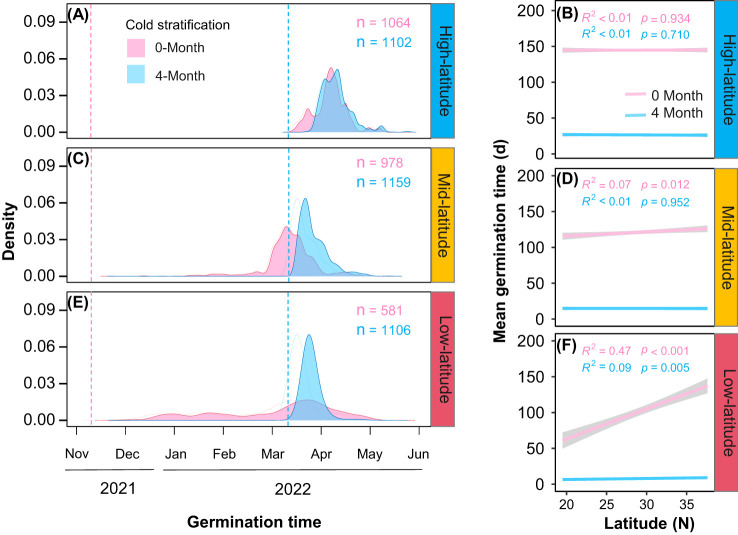
Density ridge of germination timing under different stratification time (pink for 0-month, blue for 4-month) in three common gardens (**A**: high-latitude; **C**: mid-latitude; **E**: low-latitude), sowing date marked with dashed lines. Relationships between mean germination time with latitude of origin under different stratification time in three common gardens (**B**: high-latitude; **D**: mid-latitude; **F**: low-latitude). Shaded area indicates 95% CI.

Cold stratification (4-month) significantly shorten the germination time, with varying effects among the common gardens (**Figure 3**; **Table 1A**, Cold stratification: *Chisq* = 11867.15, *P*< 0.001; Garden * Cold stratification: *Chisq* = 111.86, *P*< 0.001). Besides, we found no significant relationship between mean germination time and origin latitudes for any either cold stratification treatment in the high-latitude garden (**Figure 3B**). In the mid-latitude garden, only provenances under cold stratification for 0-month exhibited positive latitudinal cline in germination time (**Figure 3D**). In the low-latitude garden, there were positive latitudinal clines in germination time for both cold stratification treatments (**Figure 3F**). Thus, we found that the interactive effect of cold stratification and garden on the germination time was significantly different among provenances (**Table 1A**, Garden * Cold stratification * Latitude: *Chisq* = 101.53, *P*< 0.001). From high- to low-latitude gardens, the difference in mean germination time of temperate provenances between cold stratification treatments was gradually magnified (**Figures 3B, D, F**).

**Table 1:** Mixed model analysis of (A) mean germination time and (B) final germination rate of *Spartina alterniflora*, common garden site, cold stratification time and latitude of seed provenance as fixed effect with (provenance) as random effect.

Factor	(A) Mean germination time			(B) Final germination rate		
	Chisq	*Df*	*P*	Chisq	*Df*	*P*
Common Garden (G)	649.44	2	**<0.001**	84.36	2	**<0.001**
Cold stratification (C)	11867.15	1	**<0.001**	107.98	1	**<0.001**
Latitude (L)	5.21	1	**0.022**	14.00	1	**<0.001**
G*C	111.86	2	**<0.001**	73.06	2	**<0.001**
G*L	96.93	2	**<0.001**	5.56	2	0.062
C*L	69.16	1	**<0.001**	1.90	1	0.168
C*G*L	101.53	2	**<0.001**	27.82	2	**<0.001**

The significant P values were shown in bold (P < 0.05).

### Variation in final germination rate

**Figure 4:**
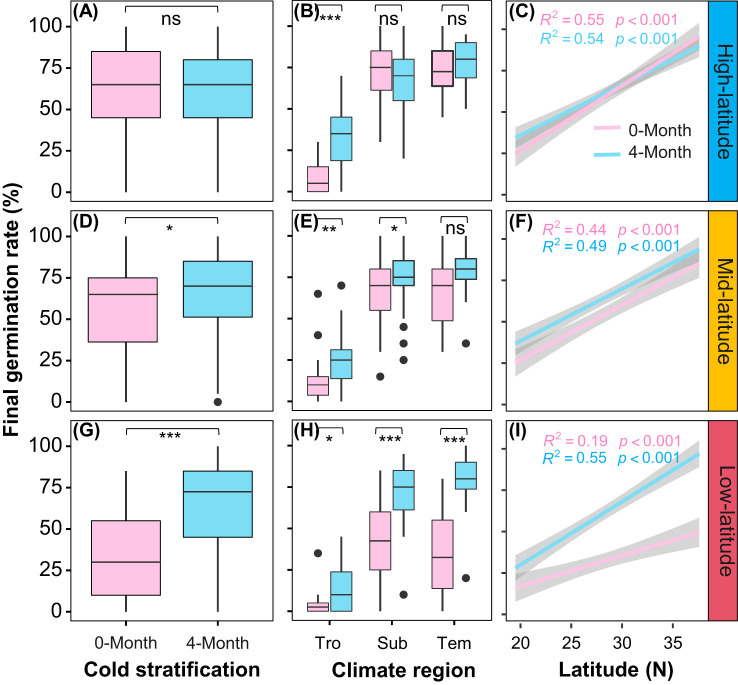
Overall average of final germination rate for nine populations under different stratification time in three common gardens (**A**: high-latitude; **D**: mid-latitude; **G**: low-latitude); average of final germination rate for seeds from different climatic zones (Tro: tropical regions; Sub: subtropical regions; Tem: temperate regions) under different stratification time in three common gardens (**B**: high-latitude; **E**: mid-latitude; **H**: low-latitude); Relationships between final germination rate with latitude of origin under different stratification time in three common gardens (**C**: high-latitude; **F**: mid-latitude; **I**: low-latitude). Shaded area indicates 95% CI. Significant levels: ns, P > 0.05; *, P < 0.05; **, P < 0.01; ***, P < 0.001.

Overall, cold stratification (4-month) improved the final germination rate and such effect was gradually magnified from high- to low-latitude gardens (**Figures 4A, D, G**; **Table 1B**, Cold stratification: *Chisq* = 107.98, *P*< 0.001; Garden * Cold stratification: *Chisq* = 73.06, *P*< 0.001). In the high-latitude garden, significant difference in final germination rate between cold stratification treatments were seen only in tropical provenances (**Figure 4B**). In the mid-latitude garden, such significant difference was observed in the tropical and subtropical provenances (**Figure 4E**). In the low-latitude garden, such significant difference was more obvious in the subtropical and temperate provenances (**Figure 4H**). Thus, we found that the interactive effect of cold stratification and garden on the final germination rate varied significantly among provenances (**Table 1B**, Garden * Cold stratification * Latitude: *Chisq* = 27.82, *P*< 0.001). Meanwhile, there were significantly positive latitudinal clines in final germination rate for both cold stratification treatments in each garden (**Figures 4C, F, I**).

## Discussion

We found that cold stratification could significantly short seeds germination time and increase germination rates of *S. alterniflora*, and increased temperature magnified the effect of cold stratification on germination. Besides, different geographic provenances of *S. alterniflora* had variable responses to the interaction of cold stratification and warming. Currently, under the climate warming, higher latitude provenances are experiencing greater warming. And their responses to both cold stratification and warming are more pronounced than other ones in this study. Therefore, such differences in germination responses among different provenances of *S. alterniflora* may lead to changes in its distribution dynamics.

### Effects of cold stratification on gemination

As the earliest life-history stage, seed germination determines individual fitness and species distribution (Donohue et al., 2005; Akiyama and Ågren, 2014). Temperature often plays a sensitive signal for seed germination (Penfield, 2008; Chamorro et al., 2018), with cold stratification (4°C) being essential for triggering germination (García-Fernández et al., 2015; Hayasaka et al., 2020). Our study found that 4-month cold stratification significantly shortened germination time and improved final germination rates (**Figures 2**–**4**), aligning with the results of a previous temperature controlled-experiment on *S. alterniflora* germination (Cheng et al., 2022). On the one hand, cold stratification boosts germination by stimulating the synthesis of hormones such as gibberellins and gibberellic acid (GA) (Deng et al., 2016; Mitchell et al., 2020). On the other hand, low temperature effectively promotes the activity of starch synthase, allowing more starch to accumulate in the seeds, providing more energy for future development. For example, Yang et al. (2019) found that in rice seeds, the ratios of GA/ABA, GA/IAA and IAA/ABA and H_2_O_2_ level were gradually enhanced with cold temperature, which might contribute to the increase of α-amylase activity to promote dormancy release in rice. The importance of cold stratification has been confirmed in Gramineae species, such as *Triticum aestivum* (Tuttle et al., 2015), *Hordeum vulgare* (Smith et al., 1996) and *Brachypodium distachyon* (Zhang et al., 2022), as well as in cosmopolitan species, like *Arabidopsis thaliana* (Nordborg and Bergelson, 1999), *Aruncus dioicus* (Giannì et al., 2019), and *Persicaria hydropiper* (Araki and Washitani, 2000). After undergoing a period of cold stratification, seeds become more sensitive to environmental cues such as temperature and humidity, enabling seeds to germinate under suitable conditions (Gremer et al., 2020a; Fernández-Pascual et al., 2021). Over four decades, *S. alterniflora* has extensively invaded China’s coasts. In its major distribution areas (temperate and subtropical regions), *S. alterniflora* is exposed to temperatures below 4°C in the winter (**Supplementary Table S2**). Our research findings indicate that *S. alterniflora* benefited from cold stratification. Consequently, indicating that low temperature in the winter may facilitate the rapid expansion of *S. alterniflora* along the Chinese coast.

### Warming altered the effects of cold stratification on germination

Temperature is a key factor in controlling seed germination characteristics (Baskin and Baskin, 1988; Kuroda and Sawada, 2022). Cold stratification at low temperature is necessary for seeds of some species to break dormancy. Under climate warming, the seed germination would be affected, e.g., delayed, because the shortened winters may not adequately overcome dormancy (Walck et al., 2011). We found that cold stratification treatment significantly led to a rapid burst of germination and improved the final germination rates. Studies focused on species that distribute along wide altitudinal gradient have shown a positive relationship between the cold stratification and the maximum seed germination (Cavieres and Sierra-Almeida, 2018; Cheng et al., 2022). Cold stratification can effectively reduce the temperature requirement for germination (Fernández-Pascual et al., 2019), thus trigger germination easily. Besides, our results suggested that the effect of cold stratification on germination of *S. alterniflora* was magnified with rising temperature (**Figures 2**–**4**; **Table 1**). Typically, higher temperatures during the germination process may enhance the physiological metabolism of plant individuals, thereby accelerating the germination (Deng et al., 2016; Mitchell et al., 2020). For example, research on the perennial herbaceous plant *Ludwigia hexapetala*, which is widely invasive in North American aquatic ecosystems, found that high temperatures caused seeds to germinate earlier (Gillard et al., 2019). This finding provides important clues to understanding plant germination in response to climate warming. As *S. alterniflora* extended southward in the past decades, higher temperature during the germination process may intensify the impact of cold stratification on its germination, facilitating its spread in lower latitude regions.

### Effects of cold stratification and warming on the germination varied among provenances

Plant germination characteristics vary across populations, influenced by genetic and environmental factors (Penfield, 2008; Walck et al., 2011; Spindelböck et al., 2013). Seeds native to high-latitudes populations may be adapted to colder temperature compared to low-latitude ones. Our hierarchical analysis verified that the seed’s origin climatic condition primarily controls the final germination rate of *S. alterniflora*, showing local adaptation to low-temperature in provenances from temperate regions (**Figures 4C, F, I**; **Supplementary Figure S2B**). This local adaptation in early germination stage has been found in various plants like *Tragopogon pratensis* (Jorritsma-Wienk et al., 2007), *Haloxylon ammodendron* (Huang et al., 2003) and *Arabidopsis thaliana* (Vidigal et al., 2016). Early life history stages are more susceptible to local adaptation due to strong natural selection (Lloret et al., 2004; Donohue et al., 2005).

Besides, our results showed that the effects of cold stratification and warming on the seed germination characteristics were more pronounced in the provenances from higher latitudes. As the decreased latitude of the common gardens, the extremely high temperature in the low-latitude garden could be stressful for *S. alterniflora* (Chen et al., 2023), the final germination rate of all provenances (under 4°C for 0-month) was significantly reduced (**Figures 4B, E, H**; **Table 1B**). This finding was consistent with a previous study on the germination differences between nonnative and native species (Hou et al., 2014) The duration of cold temperatures in winter in the tropical region may be not cold enough to break dormancy, therefore, the capacity of germination of seeds could be drastically reduced (García-Fernández et al., 2015; Cuena-Lombraña et al., 2020). Meanwhile, we found that seeds of subtropical and temperate provenances (under 4°C for 4-month) maintained consistently high germination rates across different gardens. This suggests that cold stratification may alleviate the negative effect of high temperature on germination. In the scenario of climate warming, the higher latitude regions are projected to experience greater warming than other regions (Cohen et al., 2014; Leblans et al., 2017). Many studies have reported on the effects of warming on seed germination in arctic and alpine areas (Bernareggi et al., 2016; Cavieres and Sierra-Almeida, 2018; Chamorro et al., 2018). Therefore, it is anticipated that the constraint of germination at high latitudes due to rising temperature in the future may lead to the northward migration of *S. alterniflora* populations, in an effort to locate suitable low-temperature habitat for cold stratification. For the tropical provenances, however, warming also suppressed the advantage that cold stratification provides in enhancing the germination rate, which may hinder their further spread southward. Additionally, the differences in germination responses among different provenances suggested the potential for local adaptation.

## Conclusions

Our study elucidated the considerable effect of cold stratification treatment on the seed germination of *S. alterniflora* along latitude. Meanwhile, we found that with the decreased common garden latitude (increased temperature), the effect of cold stratification on seed germination was magnified. These findings offer insight into the responses of germination in the scenario of climate warming. Plants inhabited higher latitudes are subjected to greater extent of warming contemporarily, and our results suggested that higher latitude provenances had greater responses to the interaction of cold stratification and warming. Cold stratification alleviated high temperature’s negative effects on germination, suggesting these provenances might migrate northward in search for adequate low-temperature environments. However, relying solely on common garden experiments has limitations, especially in simulating natural conditions and predicting the long-term impacts of climate change on invasive species. Although common garden experiments can control environmental variables effectively, they do not fully capture the complexity of ecological and climate fluctuations in natural habitats. Therefore, integrating observational studies, experimental warming methods and common garden experiments are the promising way to advance the understanding of species responses to climate warming (Frenne et al., 2013). Overall, our study contributes to understanding the recruitment and distribution dynamics of widespread plant species and the management of exotic species in the context of climate warming.

## Data Availability

The raw data supporting the conclusions of this article will be made available by the authors, without undue reservation.
