# Supplementary material for: Warming altered the effect of cold stratification on the germination of Spartina alterniflora across climatic zones in its invasive range
Source: Front Plant Sci. 2024 Nov 26;15:1491275. doi: 10.3389/fpls.2024.1491275 (PMC11628297; doi:10.3389/fpls.2024.1491275)
Supplement: Supplementary file 1 [file Table1.docx]

***Supplementary Materials***

**Supplementary results**

Cold stratification (4-month) significantly shorten the T_90%_ (The number of days to 90% of final germination rate). The T_90%_ (average across all seeds from different provenances) were 127, 135, and 155 when under cold stratification (0-month), whereas 12, 25, and 36 of the T_90%_ were detected under cold stratification (4-month) in the low-, mid-, and high-latitude gardens, respectively (Figure S1).

The hierarchical partitioning results indicate that mean germination time was more strongly influenced by the common garden's environmental temperature during germination after cold stratification treatment, with the effect of average maximum temperature (T_max_) being the highest, with an independent effect of 23.46% (Figure S2a). In contrast, the environmental temperature of the seed provenance origin is the primary environmental factor controlling the final germination rate, with the effect of annual maximum temperature (T’_max_) remaining the highest even after cold stratification treatment, with an independent effect of 32.05%. Furthermore, after cold stratification treatment, the explanatory power of the common garden environmental temperature during germination decreased, with the independent effects of all individual climate factors except annual active accumulated temperature (T’_accum_) being less than 1% (Figure S2b).

**Supplementary tables**

**Supplementary Table 1.** The geographic locations and sowing date of *Spartina alterniflora* provenances in common gardens.

| **Locations** | **Latitude**  **(°N)** | **Experimental treatment date** |  | **Sowing date**  **(cold stratification 0-month)** | | |  | **Sowing date**  **(cold stratification 4-month)** | | |
| --- | --- | --- | --- | --- | --- | --- | --- | --- | --- | --- |
|  |  |  |  | **Zhanjiang** | **Taizhou** | **Dongying** |  | **Zhanjiang** | **Taizhou** | **Dongying** |
| Dongying | 38 | 2021/11/10 |  | 2021/11/11 | 2021/11/10 | 2021/11/11 |  | 2022/3/11 | 2022/3/09 | 2022/3/13 |
| Ganyu | 35 | 2021/11/10 |  | 2021/11/11 | 2021/11/10 | 2021/11/11 |  | 2022/3/11 | 2022/3/09 | 2022/3/13 |
| Rudong | 32 | 2021/11/10 |  | 2021/11/11 | 2021/11/10 | 2021/11/11 |  | 2022/3/11 | 2022/3/09 | 2022/3/13 |
| Fengxian | 31 | 2021/11/10 |  | 2021/11/11 | 2021/11/10 | 2021/11/11 |  | 2022/3/11 | 2022/3/09 | 2022/3/13 |
| Yueqing | 28 | 2021/11/10 |  | 2021/11/11 | 2021/11/10 | 2021/11/11 |  | 2022/3/11 | 2022/3/09 | 2022/3/13 |
| Luoyuan | 27 | 2021/11/10 |  | 2021/11/11 | 2021/11/10 | 2021/11/11 |  | 2022/3/11 | 2022/3/09 | 2022/3/13 |
| Yunxiao | 24 | 2021/11/10 |  | 2021/11/11 | 2021/11/10 | 2021/11/11 |  | 2022/3/11 | 2022/3/09 | 2022/3/13 |
| Leizhou | 21 | 2021/11/10 |  | 2021/11/11 | 2021/11/10 | 2021/11/11 |  | 2022/3/11 | 2022/3/09 | 2022/3/13 |
| Danzhou | 20 | 2021/11/10 |  | 2021/11/11 | 2021/11/10 | 2021/11/11 |  | 2022/3/11 | 2022/3/09 | 2022/3/13 |

Note: Zhanjiang (low-latitude common garden); Taizhou (mid-latitude common garden); Dongying (high-latitude common garden).

**Supplementary Table 2.** The geographic locations and climate of nine populations of *Spartina alterniflora* were used in this study. Location name and latitude for each sampling locations on the coast of China. Climate data included mean annual temperature (T_mean_), mean annual maximum temperature (T_max_), mean annual minimum temperature (T_min_), annual number of growing degree days (AGDD), and range of days of mean daily temperature below 4 °C (D_below 4_ _°C_) for the long-term averages in 2011-2021 in field for each population.

| **Climatic regions** | **Provinces** | **Locations** | **Latitude**  **(°N)** | **T_mean_(°C)** | **T_max_(°C)** | **T_min_(°C)** | **AGDD**  **(≥ 10 °C)** | **D_below 4 °C_(d)** |
| --- | --- | --- | --- | --- | --- | --- | --- | --- |
| Temperate | Shandong | Dongying | 38 | 13.1 ± 0.2 | 33.5 ± 0.5 | -9.1 ± 0.7 | 4412.1 ± 47.2 | 64-114 |
|  | Jiangsu | Ganyu | 35 | 14.5 ± 0.3 | 36.6 ± 0.2 | -10.3 ± 0.8 | 4886.2 ± 49.2 | 50-102 |
| Subtropics | Jiangsu | Rudong | 32 | 15.8 ± 0.2 | 36.6 ± 0.2 | -7.1 ± 0.6 | 5241.3 ± 47.2 | 31-70 |
|  | Shanghai | Fengxian | 31 | 17.4 ± 0.2 | 37.4 ± 0.4 | -4.9 ± 0.5 | 5830.0 ± 56.6 | 8-49 |
|  | Zhejiang | Yueqing | 28 | 19.2 ± 0.2 | 37.0 ± 0.4 | -0.3 ± 0.6 | 6649.5 ± 84 | 0-8 |
|  | Fujian | Luoyuan | 27 | 20.8 ± 0.2 | 38.3 ± 0.2 | 2.5 ± 0.5 | 7413.6 ± 79.6 | 0-2 |
|  | Fujian | Yunxiao | 24 | 21.6 ± 0.2 | 36.4 ± 0.4 | 5.0 ± 0.6 | 7819.0 ± 65 | 0-1 |
| Tropics | Guangdong | Leizhou | 21 | 23.7 ± 0.2 | 35.9 ± 0.3 | 6.2 ± 0.5 | 8626.4 ± 79.7 | 0-1 |
|  | Hainan | Danzhou | 20 | 24.6 ± 0.2 | 37.9 ± 0.4 | 8.5 ± 0.5 | 8976.8 ± 75.3 | 0 |

**Supplementary Table 3.** Location and climate information for multiple common gardens. Climate data included mean daily temperature (T’_mean_), mean daily maximum temperature (T’_max_), mean daily minimum temperature (T’_min_), the number of growing degree days (GDD), and days of mean daily temperature below 4 °C (D’_below 4 °C_) during the germination period from November 2021 to May 2022 in each common garden.

| **Common garden**  **latitude** | **Provinces** | **Locations** | **Latitude**  **(°N)** | **Cold**  **stratification** | **T’_mean_(°C)** | **T’_max_(°C)** | **T’_min_(°C)** | **GDD**  **(≥ 10 °C)** | **D’_below 4 °C_(d)** |
| --- | --- | --- | --- | --- | --- | --- | --- | --- | --- |
| High | Shandong | Dongying | 38 | 0-month | 9.1 | 23.8 | 0.6 | 1471.7 | 74 |
|  |  |  |  | 4-month | 17.7 | 31.2 | 8.2 | 1349.3 | 3 |
| Mid | Zhejiang | Taizhou | 28 | 0-month | 15.0 | 26.8 | 9.3 | 2690.9 | 1 |
|  |  |  |  | 4-month | 20.6 | 34.1 | 14.0 | 1727.8 | 0 |
| Low | Guangdong | Zhanjiang | 21 | 0-month | 23.3 | 35.1 | 17.7 | 4700.2 | 0 |
|  |  |  |  | 4-month | 27.4 | 40.2 | 21.5 | 2299.3 | 0 |

Note: The climatic data of the common garden was collected from the sowing date to the end of the experiment (cold stratification 0-month: November 2021 to May 2022; cold stratification 4-month: March 2022 to May 2022). The specific cold stratification treatment date and sowing date information as shown in Table S1.

**Supplementary Figures**


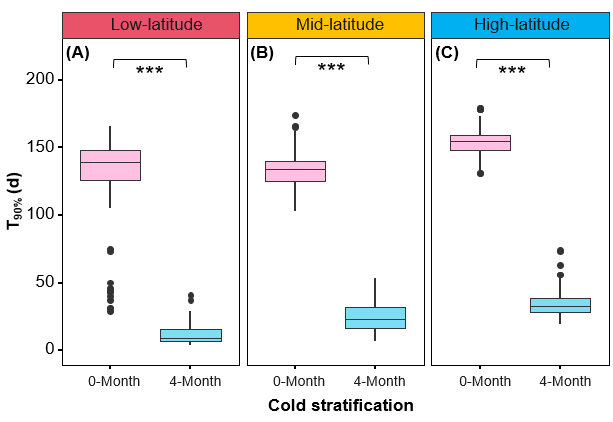


**Supplementary Figure 1.** T_90%_ (The number of days to 90% of final germination rate) of *Spartina alterniflora* under stratification time in three common gardens (a: low-latitude; b: mid-latitude; c: high-latitude). Significant levels: ***, *P* < 0.001.


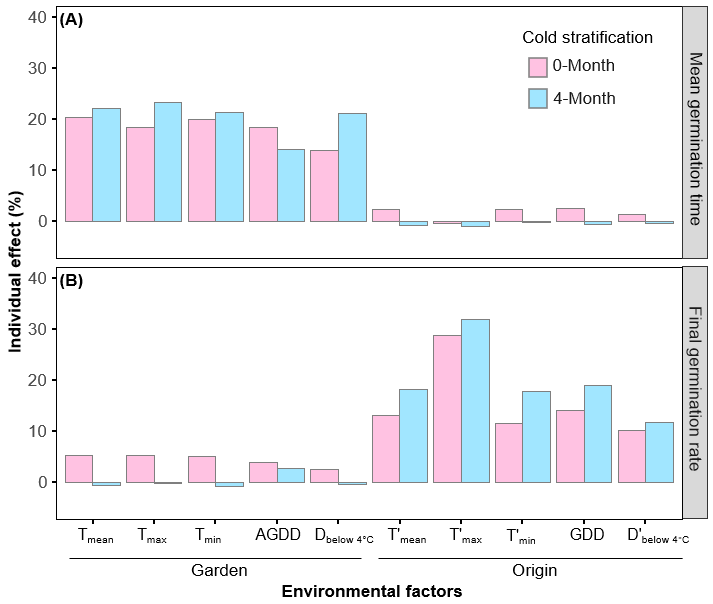


**Supplementary Figure 2.** Hierarchical partitioning analysis of environmental variables of germination character (a: final germination rate; b: mean germination time) under stratification time.
